# The Story of the Finest Armor: Developmental Aspects of Reptile Skin

**DOI:** 10.3390/jdb11010005

**Published:** 2023-01-28

**Authors:** Melodi Yenmiş, Dinçer Ayaz

**Affiliations:** Biology Department, Faculty of Science, Ege University, Bornova, 35050 İzmir, Turkey

**Keywords:** reptile, epidermis, keratin, protein, development, origin

## Abstract

The reptile skin is a barrier against water loss and pathogens and an armor for mechanical damages. The integument of reptiles consists of two main layers: the epidermis and the dermis. The epidermis, the hard cover of the body which has an armor-like role, varies among extant reptiles in terms of structural aspects such as thickness, hardness or the kinds of appendages it constitutes. The reptile epithelial cells of the epidermis (keratinocytes) are composed of two main proteins: intermediate filament keratins (IFKs) and corneous beta proteins (CBPs). The outer horny layer of the epidermis, stratum corneum, is constituted of keratinocytes by means of terminal differentiation or cornification which is a result of the protein interactions where CBPs associate with and coat the initial scaffold of IFKs. Reptiles were able to colonize the terrestrial environment due to the changes in these epidermal structures, which led to various cornified epidermal appendages such as scales and scutes, a beak, claws or setae. Developmental and structural aspects of the epidermal CBPs as well as their shared chromosomal locus (EDC) indicate an ancestral origin that gave rise to the finest armor of reptilians.

## 1. Introduction

The terrestrial adaptation of vertebrates necessitated the development of a new integument with distinct characteristics from its aquatic ancestors. Therefore, a skin with protective abilities has evolved. The reptile skin acts as a barrier against water loss and pathogens, as well as an armor against mechanical damage. In the process of reaching this outcome, the epidermal layers of the integument undergo a specialized differentiation known as cornification, which increased resilience [1,2,3,4]. This puissant epidermis, which enables a multifarious appearance, makes reptilian integument the finest armor among vertebrates.

**Figure 1:**
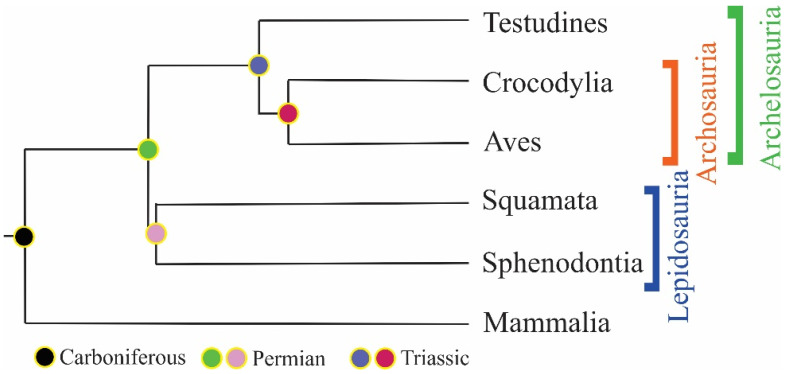
Evolutionary tree of amniotes (adapted from Ref. [5]. 2009, Shedlock, A.M., et al.).

Sauropsida clade consists of reptiles and birds excluding synapsida (extinct ancient reptiles). Extant reptiles are classified under three subclasses: Anapsida, Lepidosauria and Archosauria [5]. Recent studies are evaluating the Anapsida and Archosauria together under the clade Archelosauria [6] (Figure 1).

This review puts the structure, organization and development of the general and clade-specific reptilian epidermis, its hard appendages, and the protein components, under the scope successively with original illustrations of the mentioned characteristics to compare and state the known aspects for a better evolutionary evaluation of the terrestrial adaptation.

## 2. Evolution, the Armorer: The Cornified Epidermis

The integument of reptiles consists of two main layers: the epidermis and the dermis. The epidermis, the hard cover of the body which has an armor-like role, varies among extant reptiles in terms of structural aspects such as thickness, hardness or the kinds of appendages it constitutes. The epidermis of lepidosaurs has inner and outer generations due to the shedding phenomenon. The outer generation, that is, the one that will be shed, has six layers on end which are clear-, lacunar-, alpha-, mesos-, beta- and Oberhäutchen layers from the inside out. Archelosaurians, on the other hand, have a simpler epidermis with a laminal epithelium covered with a thick cornified layer [7,8,9,10]. 

### 2.1. Making of the Armor: Proteins

Studies have presented that the reptile epithelial cells of the epidermis (keratinocytes) are composed of two main proteins: intermediate filament keratins (IFKs) and corneous beta proteins (CBPs) [11,12].

IFKs (or cytokeratins) are 40–70 kDa, 450–650 amino acid long proteins composed of 8–12 nm filaments with an alpha-helix domain at the center (central-rod) that allows for the heterodimer structure and filament organization as well as a large part of IFK molecule. *N*- and *C*- terminals of the protein, however, are formed in beta-sheets that are normally short but can differ in length under specific stress conditions [1,13,14,15,16]. IFKs, which contain glycine and cysteine amino acids in variable quantities, made the amniote integument more suitable for the terrestrial environment. They are encoded by chromosomal loci, the number of which varies according to the species. IFK genes that have eight to nine exons form two types of clusters: acidic Type I and basic Type II. One of each type of keratins come together to form a dimer and after that they generate a tetramer, a protofilament and the IFK filament, respectively. IFKs are the main fibrous and cytoskeletal proteins generated by the basal and suprabasal layers of the epidermis as well as the skin appendages [17,18,19,20,21]. 

**Figure 2:**
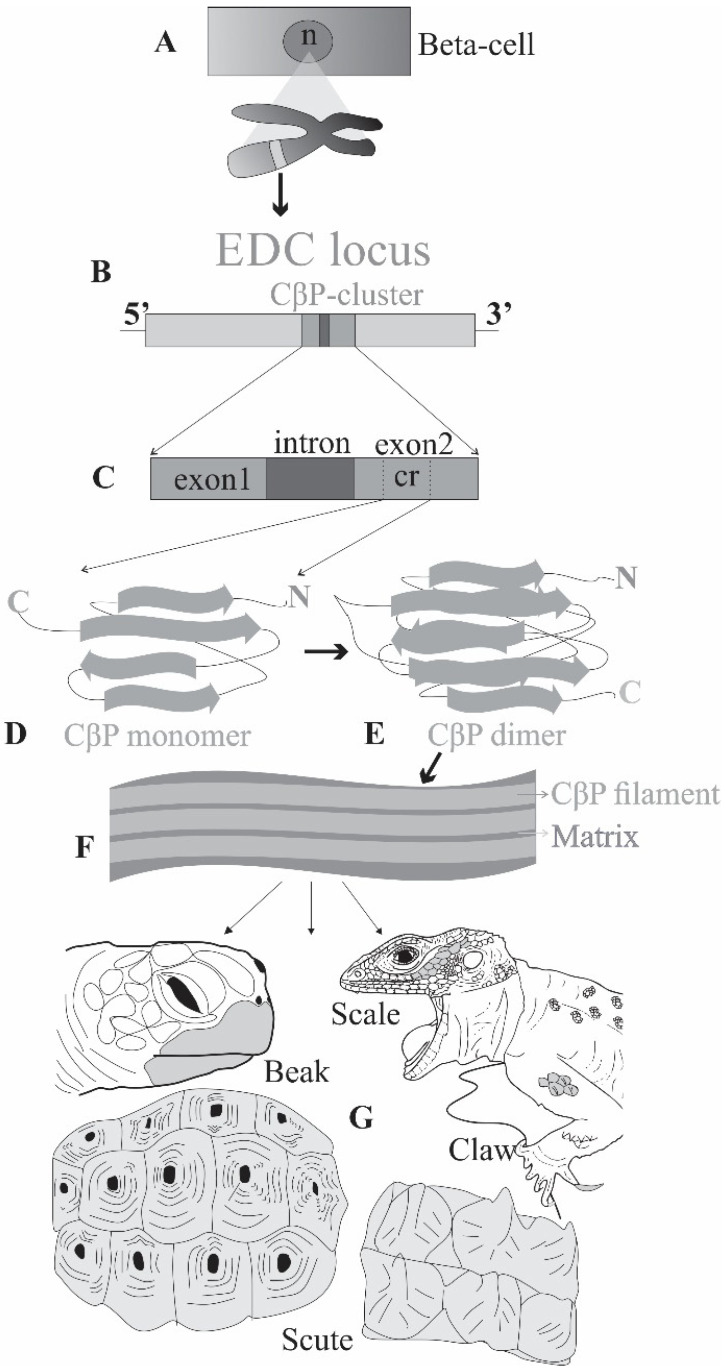
The formation of CBP (**A**) Chromosomal EDC locus within the nucleus (n) of a beta-cell. (**B**) The CBP cluster within the EDC locus. (**C**) Closer look to CBP cluster showing one intron and two exons of which only the second one has a coding region (cr). (**D**,**E**) Monomer and dimer of CBP, respectively, showing beta-sheet conformation. (**F**) CBP filaments covered and inter-filamentous matrix. (**G**) Different horny epidermal appendages made of CBPs, such as beak, scale, claw and scutes (The figure is modified from [20], Reprinted/adapted with permission from Ref. [20]. 2016, Elsevier).

CBPs, on the other hand, are 70–240 amino acid long proteins 8–30 kDa proteins with a 34-amino acid center of which 20 have a high homology (core-box) in beta-sheet form that generates 3–4 nm thick filaments [22,23,24]. Core-box is an evolutionary formation unique to sauropsids [21,25,26,27]. For the synthesis of CBPs, lizards have 40–75 genes, while there are 40–90 genes in turtles and 20–30 genes in crocodilians [28,29,30]. CBPs are a subtype of corneous protein family (CPs) of the amniotes and are coded from a major sub-cluster that comprises one intron and two exons (first non-coding and the second coding) of Epidermal Differential Complex (EDC, see below) locus among many other corneous proteins (e.g., filaggrin, loricrin, involucrin, cornulin, and S100 proteins [26,27,31]. The chromosomal position of the CBP coding EDC is distant from those alpha-keratin coding genes [32]. The hierarchical organization of the CBP molecule begins when two monomer proteins come together and form a dimer (beta-sandwich) which is the main constituent of the beta-filaments. These filaments undergo beta-sheet folding to make the CBP molecule which will be the building stone of the firm horny layer, stratum corneum of the epidermis. This cornification creates a rigid armor capable of movement rather than complete rigidity due to the density of the proteins and the thickness of the horny layer and is sometimes even involved in the softer epidermis of some turtles, pliable-but-resistant layer of snakes and lizards and the formation of flexible appendages, such as lizard adhesive setae [11,33] (Figure 2).

Different reptiles have varying CBPs in terms of the longevity and the amino acid structure of the N- and C- terminals even though the core-box of the protein is highly conserved among the taxa [3,34,35]. One of the amino acids at the terminals is cysteine, which constitutes 4–20% of the composition that allows the formation of disulfide bonds. Other than cysteine, there are glycine and tyrosine residues that have been considered as the sources of the hardness and pliancy of the epidermal appendages [35,36].

There is no CBP synthesis in the basal layer of the epidermis; rather, it is produced in the suprabasal layers, which are the only sites where genes are active, as are other EDC proteins. CBPs are added to the cytoskeleton formed by the IFKs within the corneocytes only later, in the upper layers of the epidermis, where the initial mass of the IFKs is 30–60% of the total proteins. When this percentage starts to express the CBP mass after the interaction, it means that the skin is cornified [37,38,39,40,41].

### 2.2. Hardening the Armor: Cornification through Protein Interactions 

The outer horny layer of the epidermis, stratum corneum is constituted of keratinocytes by means of terminal differentiation or cornification, which is a result of the protein interactions where CBPs associate with and coat the initial scaffold of IFKs. This process paved the way for vertebrates in the harsh and dry conditions of terrestrial environments during the Carboniferous and Permian periods, when hyper-keratinization turned into cornification [12,20,21,23,42].

The interaction process among IFKs and CBPs (e.g., type, ratio etc.) varies among the taxa and determines the aspects of the mature corneous layer in terms of water relations (hydrophobic/hydrophilic structure), flexibility, or filamentous pattern. The process from keratinization to cornification, which took place during the skin evolution of vertebrates, occurred with the evolution of IFKs, CBPs and CPs (corneous proteins of the mammalian), respectively [14,21]. 

Basal IFKs formed the cytoskeleton, while suprabasal ones enriched it. It formed tonofilaments on its way to the skin surface and eventually made keratinization with keratinocyte deaths. In the evolutionary process, the amino acid content of IFKs has changed, the amount of glycine, which regulates water relations, and the amount of cysteine, which increases filling/packing by increasing SS bonds, has increased. In addition, the cross-links between the keratin filaments were strengthened to increase the hardness. As a result of the interaction (masking or degradation) of IFKs and CBPs in the upper layers of the skin, terminal differentiation occurred, and with the death of corneocytes (corneoptosis, a unique mode of cell death) the skin became cornified with the resistant, covalent bonds, thus gaining the ability to prevent water loss, microbial diseases, and mechanical injuries [35,43,44,45]. The process from keratinization to cornification can be observed in amniotic embryos, where the embryonic epidermis of the skin appendages accumulates keratins in the first place, but the maturation takes place by the jointure of a high portion of CBPs [41,46]. 

## 3. Diversifying the Armor: Epidermal Appendages

Reptiles have various cornified epidermal appendages such as scales and scutes, a beak, claws, or setae. Here, we approach them in a specialized manner in terms of their developmental aspects and general structures.

### 3.1. Turtle Scutes

Placodes are the source of archosaur and anapsid scutes. However, the scales of Lepidosaurs, unlike these, developed without placodes [47]. The shell appendages of turtles are divided into scutes and tubercles. Scutes are also divided into two according to their cornification status. These are soft scutes with soft cornification and hard scutes with hard cornification. About 200 CBPs detected in turtles are divided into TypeA and TypeB according to their amino acid sequences. TypeA CBPs were encoded by EDC, while others translocated out of EDC. However, both types of CBP have been found to be phylogenetically related to each other. In some studies, CBPs encoded by EDC were thought to be diverse and more numerous due to taxon-specific skin appendages. However, other studies have found more EDC-coded CBPs in soft-shelled turtles than in hard-shelled ones, reopening the previous idea [29,48,49].

The first indication of shell formation during the embryonic development of the turtle is the formation of the carapacial ridge. The scutes formed by the interaction of ectoderm and mesenchyme and by changing and expanding the surfaces of the placodes follow the ridge. Scales and tubercles develop last [50,51].

Shell formation is a proximodistal growth, that is, different structures evolve simultaneously from the center to the outside or vice versa. The order of formation of carapace scutes is marginal, costal, vertebral, and nuchal; that is, they develop from the outside to the center. Plastral ones are formed symmetrically in the periphery. There are furrows separating the scutes on the shell, those separating the costals develop from the center outward and those separating the marginals develop from the outer to the center [52,53,54,55,56].

Although turtle scutes share developmental characteristics with other epidermal appendages, they have been determined to be evolutionary different from others due to their novelties such as non-EDC encoded CBPs, proximodistal growth and carrying soft and hard scutes [47].

### 3.2. Turtle Beak (Rhamphotheca)

The turtle’s beak is a cornified skin appendage that covers the maxilla and mandible and replaces teeth. It enables to cut, crumble, divulse and clutch. The embryonic development begins with the mesenchymal condensations associated with epidermal placodes which trigger proliferation and differentiation of corneocytes. In the early stages of development, the dorsal beak develops from the frontal and maxillary placodes. At this stage, the genes that will enable CBP synthesis and accumulation are inactive. New corneocytes produce CBPs to form the maxillary and mandibular cornified components of the beak. The ventral beak develops from posteriorly enlarging mandibular placodes. Cell proliferation is lower in placodes than in scales and their hinge regions. Therefore, with the enlargement of the dorsal region, the maxillary placode develops more anteriorly than the mandible, similar to that in birds, and the difference between the beaks emerges. This similarity with birds suggests that there are homologous signaling pathways in beak formation in the two taxa [43,44,57,58,59,60,61,62]. 

Mesenchymal densities, which are initially discontinuous in the beak formation region, turn into a layer covering the jaw bones in later stages. This results in the formation of a cornified layer containing CBP. This explains the formation of cornified layers in the beak at much earlier embryonic stages than in scales, as in the claw. A total of 6–8 layers of epidermis are formed which will be shed in-ovo. After the shedding plane becomes clear, a layer of 3–4 rows of keratinocytes are formed under it. Before hatching, the contents of these cells are replaced by CBPs, this is called definitive terminal differentiation of keratinocytes. As a result, the beak content takes its hard and strong form with the addition of inorganic elements [63,64,65].

### 3.3. Lizard Setae

The structures that enable some lizards such as geckos and anoles lizards to walk or stand on low-friction surfaces such as glass easily are the setae, which are microscopic extensions formed by the differentiation of the epidermal layers on the fingertips. These setae are actually adhesive structures formed as a special shedding plane and developed from the Oberhäutchen and clear layers of the epidermis. These structures, consisting of digital pad lamellae, can be 1–4 µm in diameter and 10–120 µm long, depending on the species. They contain both 8–22 kDa CBPs and 45–60 kDa IFKs. The CBPs they carry may be rich in cysteine or glycine. These proteins allow the setae to have a cornified layer that is both flexible and resistant. This is a feature that prevents damage to the epidermis during movement. The setae are divided at their ends into much finer hairy appendages of 0.5–3 um long called spatulae, which are formed by cytoskeletal organization and mechanical separation of keratin bundles [66,67,68,69].

The extensive production of cytoskeletal proteins other than CBPs and IFKs, such as actin and tubulin, required for setae formation and development is dependent on the intense protein production during shedding of the pad lamellae. These proteins can initiate the aggregation of micro-filaments and -tubules, which are the source of cell extensions. In particular, the localization of actin and RhoV proteins indicated that they are associated with cetae formation and growth. Some microtubules are arranged spirally, forming a cytoskeleton, surrounding the growing setae and directing the growing setae into the clear layer cells. Although the number of genome-related studies is scarce, genetic structure can be pointed out as the source of this specificity, since the morphological diversity of these setae and spatulae with terminal extensions is species-specific [14,24,69].

### 3.4. Claw

Reptile claws are important epidermal appendages that have evolved to facilitate movement on the hard substrate of the terrestrial environment during the transition from water to land. The cornification on reptile claws is extremely hard. Apart from the main CBPs that make up the structure of the claw, it also contains different types of proteins. Studies have found proteins rich in glycine, cysteine, tyrosine and lysine. In this context, they have similar features to the claws of birds and the nails of mammals. Similar to the formation of scales, CBPs are produced in corneocytes. However, unlike the syncytium formed by the corneocytes in the scales, the corneocytes in the claw do not fully coalesce. Another difference is that the CBPs in the reptilian claw undergo an elongation process. Accordingly, the long cornified bundles are deposited in a regular or irregular manner and then elongate along the plane of the finger, parallel to the epidermis, similar to the mammalian nail. When CBPs in the reptilian claws are compared in terms of amino acid richness, serine, tyrosine, glycine and arginine are seen in crocodiles, cysteine in turtles, and glycine-cysteine in lizards. In addition to all this protein content, it is possible that inorganic substances such as calcium accumulate in the claw, increasing its hardness. The elongated corneocytes fuse distally to form the pointed apical of the claw, becoming hard and resistant. Unlike the epidermis of scales, a shedding plane does not form in the claw, instead, it continues to accumulate with keratinocyte, forming a compact structure called the lamina unguis [70,71,72,73,74].

Claw morphology is curved. The dorsal part of the claw is called the unguis and the ventral part is called the subunguis. Subunguis is covered by a thinner corneous layer than the unguis. What gives the claw this curve is that the cell proliferation in the dorsal region is more intense than in the ventral region. Therefore, the dorsal region grows faster and curves over the slow growing region, giving the hard claw functionality for actions such as scraping, tearing, climbing [60,75,76].

## 4. Armoring in Progress: Development of the Horny Epidermis 

In reptiles, the embryonic epidermis is formed by differentiation of the ectoderm, which forms the periderm and the basal layer [7,77]. The embryonic epidermis consists of 2 layers of periderm and 1–6 layers of transition layers. This can also be called the temporary epidermis. In the inner layer of the periderm, there are embryonic organelles made of irregular filaments 25–50 nm thick. Individuals about to hatch have CBPs rich in glycine and glycine-cysteine in the outer layer. Both layers of the periderm contain acidic IFKs, which were basic when synthesized from the ribosome but subsequently changed. This may indicate that they interact electrostatically with CBPs found only in the lower layers of the embryonic epidermis similar to adult individuals [68,78,79].

Lepidosaurs have 35–71 CBP genes. In the early stages of the lizard embryo, the three-layered epidermis consists only of IFKs. CBP activity begins 1–2 weeks before hatching. With this cornification activity, the filaments of the reticulated embryonic organelles in the periderm disappear by mixing with the keratin filaments. The final differentiation of CBP occurs by molting, which takes place in the egg before hatching [80,81]. In snakes, the embryonic epidermis is initially covered by the periderm, under which the Oberhäutchen is formed. Glycine-rich CBPs are synthesized 10–15 days before hatching. It has been said in previous studies that the number of CBPs and the diversity of the genes encoding them can be associated with the diversity of the skin derivatives of the taxon. Added to this, however, is the information that the number of CBP genes in snakes is similar to that of lizards, despite losing their members and associated skin derivatives (*A. carolinensis*: 40 genes, [22]; *P. bivittaus*, 35 genes and *O. hannah*, 36 genes, [82]). Chelonians have 37–89 CBP genes and Archosaurs have the least number of CBPs with 20–21 types. The multi-layered epidermis of the reptile embryos is formed rather recently, (e.g., stages 22–24 and stage 24 in turtles and crocodiles, respectively), but when it does, it enables the epidermal layers to differentiate. However, in the end, the horny layer that will keep the individual unharmed under terrestrial conditions would be ready before hatching [29,48,83,84,85,86]. The “living fossil” tuatara, the only extant member of the Rynchocephalia order, differs from its “classmates” by not having an Oberhäutchen and a clear layer in the stratified epidermis, by having two different types of CBPs in terms of B-sheet conformation and by the number of some shared proteins (such as SFTPs) that are encoded in the course of embryonic development. It has 20 CBP types of 16–20 kDa and IFKs of 40–63 kDa. It also has shared features, such as having more than 50 EDC genes that resemble those of other Lepidosaurian EDCs [87].

### Renovating the Armor: Shedding Cycle

**Figure 3:**
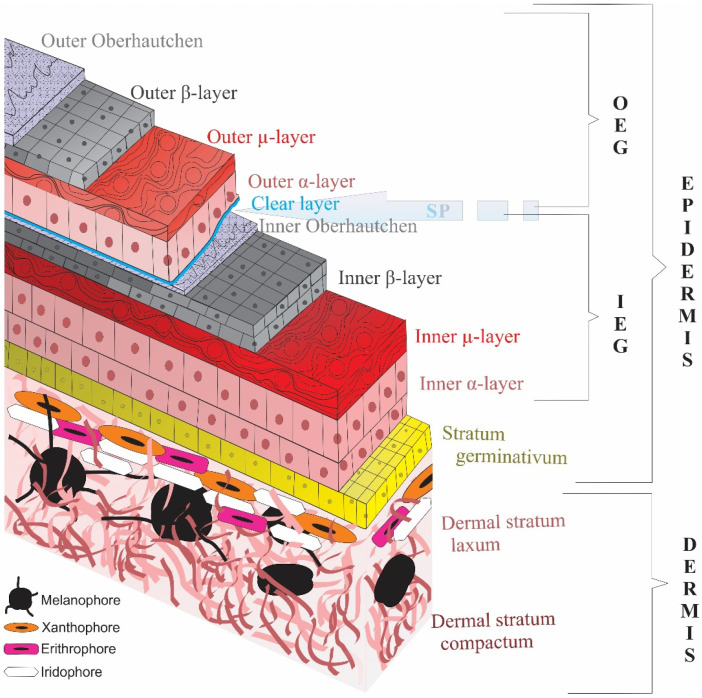
A general illustration showing layers of lepidosaurian skin. The Shedding Plane (SP) is separating the inner (IEG) and the outer epidermal generation (OEG) apart. Oberhäutchen, beta (β), mesos (µ) and alpha (α) layers are present in both generations. However, the outer generation also has a clear layer between the outer alpha and inner Oberhäutchen layers at the time of shedding. Dermis contains chromatophores giving lepidosaurians multifarious appearances (Adapted from Yenmiş, 2022 [88]).

The shedding phenomenon, which is the cyclical epidermal cast, occurs in toto in Serpentes and in fragments in Sauria and Rhyncocephalia to enable somatic growth, distinguishing Lepidosaurs from other reptiles, namely Archelosaurs. To accomplish this, the epidermis divides into two generations: the outer generation, which will be shed, and the inner generation, which will replace the former and form a shedding plane within the corneous layer through corneocyte differentiation (Figure 3). Among different stages of the shedding cycle, the outer and inner generations differentiate in terms of thickness and structure. Correspondingly, it has been hypothesized that the cornified substance of the epidermis would vary throughout the cycle. Indeed, studies have shown that CBP is absent during the resting phase and present during the regenerative stages of its expression [9,22].

Related to this, specific glycine-cysteine-rich CBPs have been found in the Oberhäutchen layer of some reptilians, indicating the formation of the shedding plane, e.g., the Sn-cys-1 protein, begins to form in the embryonic Oberhäutchen layer in the snake embryo in preparation for in-ovo shedding, which takes place 1–2 weeks before hatching. The absence of this protein in other Lepidosaurs and Archelosaurs indicates that this CBP is specific for the snake shedding cycle [2,75,89].

## 5. History of the Armor: Origin and Evolution

IKSs are present in invertebrates and chordates and the central domain of these proteins is thought to have formed one billion years ago. The first amniotes of the Carboniferous that had IFKs had evolved a different locus than IFK genes, which is called the EDC. All the vertebrates have both IFK and EDC loci. Yet the gene clusters within are clade specific. CBPs that are sauropsid-specific proteins evolved within the sauropsid EDC. The core-box of the CBPs is highly conserved among sauropsids and even the translocated locus of Chelonian-Avian claw CBPs are phylogenetically related to EDC genes, which led the researchers to suggest an ancestral EDC locus as a precursor [26,90,91,92].

Reptiles were able to colonize the terrestrial environment due to the changes in the epidermal structures, and the initial adaptation for this was the movement of the keratinocytes from the basal layer to the upper epidermal layers. After that, CBPs became variable and high in number and started to mask the IFKs which led to the coverage of corneocytes throughout the body, that is, cornification, as opposed to fish and amphibians [77,78].

The first epidermal appendage on land was probably the claw, in which hard cysteine-rich IFKs evolved. Corneous tubercles followed them in the Permian-Triassic, and with the interaction of both of these appendages, scales and rhamphotheca emerged [93,94,95,96,97].

Lepidosaurian CBPs diverged from Archosauria 278 mya which was followed by the evolution of four main CBP subtypes 70 mys later. Divergence Archosaurian-Chelonian CBPs occurred around 229 mya and turtle CBPs shaped the two types (Type I and II) after 60 mys [98].

The four avian CBPs are orthologues to four crocodile and four turtle CBP genes and share a similar organization within EDC (synteny), which points to a common Archelosaurian ancestor after the split from the Lepidosaurian branch. Besides, two other keratinocyte CBP clusters (apart from the former four) are conserved among Archelosaurs, also pointing to the common ancestor. It is thought that this root Archelosaur had 18 keratinocytes and 12 claw CBPs [3,29].

Archelosaurs split into Archosaurians and Chelonians during the Permian period. In chelonians Type I CBP (BetaA, claw subfamily) and Type II (BetaB, keratinocyte subfamily) emerged, among which the former translocated outside EDC, involved in shell formation with duplication and diversification, while the latter expanded and covered some parts of non-shelled body regions [3,28,88].

In Archosaurs similar claw and scale subfamilies diverged, and claw CBPs started to duplicate and diversify. Avian feather CBPs emerged in the Jurassic and expanded. Today, Archosaurs lost their claw subfamilies, and while they have the lowest number of CBPs, other types of EDC genes expanded. On the other hand, Lepidosaurians which includes the most variable members, have multifarious skin appendages that could be the explanation of high number for the CBPs they have [3,74].

## Data Availability

The authors confirm that the data supporting the findings of this study are available within the article.

## References

[1] Eckhart L., Lippens S., Tschachler E., Declercq W. (2013). Cell death by cornification. Biochim. Biophys. Acta.

[2] Alibardi L. (2014). Transition from embryonic to adult epidermis in reptiles occurs by the production of corneous beta-proteins. Int. J. Dev. Biol..

[3] Holthaus K.B., Eckhart L., Dalla Valle L., Alibardi L. (2020). Review: Evolution and diversification of corneous beta-proteins, the characteristic epidermal proteins of reptiles and birds. J. Exp. Zool..

[4] Akat E., Yenmiş M., Pombal M.A., Molist P., Megías M., Arman S., Veselỳ M., Anderson R., Ayaz D. (2022). Comparison of vertebrate skin structure at class level: A review. Anat. Rec..

[5] Shedlock A.M., Edwards S.V., Hedges S.B., Kumar S. (2009). Amniotes (Amniota). Timetree of Life.

[6] Crawford N.G., Parham J.F., Sellas A.B., Faircloth B.C., Glenn T.C., Papenfuss T.J., Henderson J.B., Hansen M.H., Simison W.B. (2015). A phylogenomic analysis of turtles. Mol. Phylogenet. Evol..

[7] Maderson P.F., Gans C., Billett F., Maderson P.F.A. (1985). Some developmental problems of the reptilian integument. Biology of the Reptilia.

[8] Landmann L., Bereiter-Hahn J., Matoltsy A.G., Sylvia-Richards K. (1986). The skin of Reptiles: Epidermis and dermis. Biology of the Integument.

[9] Maderson P.F.A., Rabinowitz T., Tandler B., Alibardi L. (1998). Ultrastructural contributions to an understanding of the cellular mechanisms involved in lizard skin shedding with comments on the function and evolution of a unique lepidosaurian phenomenon. J. Morphol..

[10] Alibardi L., Maderson P.F.A. (2003). Observations on the histochemistry and ultrastructure of the epidermis of the Tuatara, *Sphenodon punctatus* (Spehnodontida, Lepidosauri, Reptilis): A contribution to an understanding of the lepidosaurian epidermal generation and the evolutionary origin of the squamate shedding complex. J. Morphol..

[11] Sawyer R.H., Glenn T., French J.O., Mays B., Shames R.B., Barnes G.L., Rhodes W., Ishikawa Y. (2000). The expression of beta (b) keratins in the epidermal appendages of reptiles and birds. Am. Zool..

[12] Alibardi L., Toni M. (2006). Cytochemical, biochemical and molecular aspects of the process of keratinization in the epidermis of reptilian scales. Prog. Histochem. Cytochem..

[13] Alibardi L. (2006). Structural and immunocytochemical characterization of keratinization in vertebrate epidermis and epidermal derivatives. Int. Rev. Cytol..

[14] Alibardi L. (2013). Ultrastructural immunolocalization of alphakeratins and associated beta-proteins (beta-keratins) suggests a new interpretation on the process of hard and soft cornification in turtle epidermis. Micron.

[15] Fraser R.D., Parry D.A. (2009). The role of b-sheets in the structure and assembly of keratins. Biophys. Rev..

[16] Chou C.C., Buehler M.J. (2012). Structure and mechanical properties of human trichocyte keratin intermediate filament protein. Biomacromolecules.

[17] Coulombe P.A., Omary M.B. (2002). ‘Hard’ and ‘soft’ principles defining the structure, function and regulation of keratin intermediate filaments. Curr. Opin. Cell Biol..

[18] Alibardi L., Toni M., Dalla Valle L. (2007). Review. Hard keratins in reptilian epidermis in comparison to those of birds and mammals. Exp. Dermatol..

[19] Hallahan D.L., Keiper-Hrynko N.M., Shang T.Q., Ganzke T.S., Toni M., Dalla Valle L., Alibardi L. (2008). Analysis of gene expression in gecko digital adhesive pads indicates significant production of cysteine- and glycine-rich beta-keratins. J. Exp. Zool. B.

[20] Alibardi L. (2016). Review. The process of cornification evolved from the initial keratinization in the epidermis and epidermal derivatives of vertebrates: A new synthesis and the case of sauropsids. Int. Rev. Cell Mol. Biol..

[21] Alibardi L. (2022). Keratinization and Cornification are not equivalent processes but keratinization in fish and amphibians evolved into cornification in terrestrial vertebrates. Exp. Dermatol..

[22] Dalla Valle L., Nardi A., Bonazza G., Zuccal C., Emera D., Alibardi L. (2010). Forty keratin-associated β–proteins (β-keratins) form the hard layers of scales, claws and adhesive pads in the green anole lizard, *Anolis carolinensis*. J. Exp. Zool..

[23] Frazer R.D.B., Parry D.A.D. (1996). The molecular structure of reptilian keratin. Int. J. Biol. Macromol..

[24] Alibardi L. (2013). Cornification in reptilian epidermis occurs through the deposition of keratin associated beta proteins (beta-keratins) onto a scaffold of intermediate filament keratins. J. Morphol..

[25] Alibardi L., Toni M. (2007). Beta keratins of reptilian epidermis share a conserved common epitope termed the core-box. Res. J. Biol. Sci..

[26] Strasser B., Mlitz V., Hermann M., Rice R.H., Eigenheer R.A., Alibardi L., Tschachler E., Eckhart L. (2014). Evolutionary origin and diversification of epidermal barrier proteins in amniotes. Mol. Biol. Evol..

[27] Calvaresi M., Eckhart L., Alibardi L. (2016). The molecular organization of the beta-sheet region in corneous beta-proteins (beta-keratins) of sauropsids explains its stability and polymerization into filaments. J. Struct. Biol..

[28] Greenwold M.J., Sawyer R.H. (2013). Molecular evolution and expression of archosaurian beta-keratins: Diversification and expansion of archosaurian beta-keratins and the origin of feather beta-keratins. J. Exp. Zool. B.

[29] Li Y.I., Kong L., Pontig C.P., Haerty W. (2013). Rapid evolution of beta-keratin genes contributo to phenotypic differences that distinguish turtle and birds from other reptiles. Genome Biol. Evol..

[30] Liu Y., Zhou Q., Wang Y., Luo L., Yang J., Yang L., Liu M., Li Y., Qian T., Zheng Y. (2015). *Gekko japonicus* genome reveals evolution of adhesive toe pads and tail regeneration. Nat. Commun..

[31] Mlitz V., Strasser B., Jaeger K., Hermann M., Ghannadan M., Buchberger M., Alibardi L., Tschachler E., Eckhart L. (2014). Trichohyalin-like proteins have evolutionarily conserved roles in the morphogenesis of skin appendages. J. Investig. Dermatol..

[32] Vandebergh W., Bossuyt F. (2012). Radiation and functional diversification of alpha keratins during early vertebrate evolution. Mol. Biol. Evol..

[33] Ng C.S., Wu P., Fan W.L., Yan J., Chen C.K., Lai Y.T., Wu S.M., Mao C.T., Chen J.J., Ho M.R. (2014). Genomic organization, transcriptomic analysis, and functional characterization of avian a- and b-keratins in diverse feather forms. Genome Biol. Evol..

[34] Fraser R.D.B., Parry D.A.D. (2011). The structural basis of the filament-matrix texture in the avian/reptilian group of hard beta-keratins. J. Struct. Biol..

[35] Fraser R.D.B., Parry D.A. (2014). Amino acid sequence homologies in the hard keratins of birds and reptiles, and their implications for molecular structure and physical properties. J. Struct. Biol..

[36] Alibardi L. (2015). Review: Mapping epidermal beta-proteins distribution in the lizard *Anolis carolinensis* shows a specific localization for the formation of scales, pads and claws. Protoplasma.

[37] Candi E., Schmidt R., Melino G. (2005). The cornified envelope: A model of cell death in the skin. Nat. Rev. Mol. Cell Biol..

[38] Lippens S., Denecker G., Ovaere P., Vandenabeele P., Declercq W. (2005). Death penalty for keratinocytes: Apoptosis versus cornification. Cell Death Differ..

[39] Dalla Valle L., Nardi A., Alibardi L. (2009). Isolation of a new class of cysteine-glycineproline rich beta-proteins (beta-keratins) and their expression in snake epidermis. J. Anat..

[40] Dalla Valle L., Nardi A., Toni M., Emeera D., Alibardi L. (2009). Beta-keratins of turtle shell are glycine-proline-tyrosine-rich proteins similar to those of crocodilians and birds. J. Anat..

[41] Alibardi L. (2021). Vertebrate keratinization evolved into cornification mainly due to transglutaminase and sulfhydryl oxidase activities on epidermal proteins: An immunohistochemical survey. Anat. Rec..

[42] Baden H.P., Maderson P.F. (1970). Morphological and biophysical identification of fibrous proteins in the amniote epidermis. J. Exp. Zool..

[43] Alibardi L. (2020). Corneous beta proteins of the epidermal differentiation complex (EDC) form large part of the corneous material of claws and rhamphothecae in turtles. Protoplasma.

[44] Alibardi L. (2020). Cell proliferation, adhesion and differentiation of keratinocytes in the developing beak and egg-tooth of the turtle *Emydura macquarii*. Protoplasma.

[45] Murata T., Honda T., Mostafa A., Kabashima K. (2022). Stratum corneum as polymer sheet: Concept and cornification processes. Trends Mol. Med..

[46] Alibardi L. (2021). Development, structure, and protein composition of reptilian claws and hypotheses of their evolution. Anat. Rec..

[47] Moustakas-Verho J.E., Cherepanov G.O. (2015). The integumental appendages of the turtle shell: An evo-devo perspective. J. Exp. Zool. B.

[48] Dalla Valle L., Michieli F., Benato F., Skobo T., Alibardi L. (2013). Molecular characterization of alpha-keratins in comparison to associated beta-proteins in soft-shelled and hard-shelled turtles produced during the process of epidermal differentiation. J. Exp. Zool. B.

[49] Holthaus K.B., Strasser B., Sipos W., Schmidt H.A., Mlitz V., Sukseree S., Weissenbacher A., Tschachler E., Alibardi L., Eckhart L. (2015). Comparative genomics identifies epidermal proteins associated with the evolution of the turtle shell. Mol. Biol. Evol..

[50] Moustakas-Verho J.E., Zimm R., Cebra-Thomas J., Lempiainee N.K., Kallonen A., Mitchel K.L., Hamalainen K., Salazar-Ciudad I., Jernvall J., Gilbert S.F. (2014). The origin and loss of periodic patterning in the turtle shell. Development.

[51] Alibardi L., Minelli D. (2015). Sites of cell proliferation during scute morphogenesis in turtle and alligator are different from those of lepidosaurian scales. Acta Zool..

[52] Cherepanov G.O. (2005). Turtle Shell: Morphogenesis and Evolution.

[53] Cherepanov G.O. (2006). Ontogenesis and evolution of horny parts of the turtle shell. Fossil Turtle Research. Suppl. Russ. J. Herpetol..

[54] Cherepanov G.O. (2014). Patterns of scute development in turtle shell: Symmetry and asymmetry. Paleontol. J..

[55] Milinchovitch M.C., Manukyan L., Debry A., Di-Poi N., Martin S., Sungh D., Lambert D., Zwicker M. (2012). Crocodile head scales are not developmental units but emerge from physical cracking. Science.

[56] Wu P., Hou L., Plikus M., Hughes M., Scehnet J., Suksaweang S., Widelitz R., Jiang T.X., Chuong C.M. (2004). Evo-Devo of amniote integuments and appendages. Int. J. Dev. Biol..

[57] Wu P., Jiang T.X., Shen J.Y., Widelitz R.B., Chuong C.M. (2006). Morphoregulation of avian beaks: Comparative mapping of growth zone activities and morphological evolution. Dev. Dyn..

[58] Alibardi L. (2009). Development, comparative morphology and cornification of reptilian claws in relation to claws evolution in tetrapods. Contrib.Zool..

[59] Alibardi L. (2009). Embryonic keratinization in vertebrates in relation to land colonization. Acta Zool..

[60] Alibardi L. (2016). Microscopic and immunohistochemical study on the cornification of the developing beak in the turtle *Emydura macquarii*. J. Morphol..

[61] Moldowan P., Litzgus J.D., Brooks R.J. (2016). Turtles with teeth: Beak morphology of testudines with a focus on the tomiodonts of painted turtles (*Chrysemys* spp.). Zoomorphol..

[62] Alibardi L. (2002). Immunocytochemical observations on the cornification of soft and hard epidermis in the turtle *Chrysemis picta*. Zoology.

[63] Alibardi L. (2014). Immunolocalization of beta-proteins and alpha-keratins in the epidermis of the soft-shelled turtle explains the lack of formation of hard corneous material. Acta Zool..

[64] Alibardi L. (2014). Immunocytochemistry suggests that a prevalence of a sub-type of beta-proteins determines the hardness in the epidermis of the hard-shelled turtle. J. Exp. Zool. B.

[65] Maderson P.F.A. (1970). Lizard glands and lizard hands: Models for evolutionary study. Forma Functio.

[66] Russel A.P. (2002). Integrative functional morphology of the gekkotan adhesive system (Reptilia, Gekkota). Int. Comp. Biol..

[67] Toni M., Dalla Valle L., Alibardi L. (2007). Review. Beta-keratins in the epidermis of reptiles: Composition, sequence and molecular organization. J. Proteome Res..

[68] Alibardi L. (2020). Adhesive pads of gecko and anoline lizards utilize corneous and cytoskeletal proteins for setae development and renewal. J. Exp. Zool. B.

[69] Baden H.P., Kvedar J.C., Goldsmith L.A. (1983). The nail. Physiology, Biochemistry and Molecular Biology of the Skin.

[70] Gillespie J.M., Goldsmith L. (1991). The structural proteins of hair: Isolation, characterization and regulation of biosynthesis. Physiology, Biochemistry and Molecular Biology of the Skin.

[71] Rogers G.E. (2004). Hair follicle differentiation and regulation. Int. J. Dev. Biol..

[72] Alibardi L., Toni M. (2008). Cytochemical and molecular characteristics of the process of cornification during feather morphogenesis. Prog. Histochem. Cytochem..

[73] Alibardi L., Toni M. (2009). Immunocytochemistry and protein analysis suggest that reptilian claws contain small high cysteine-glycine proteins. Tissue Cell.

[74] Alibardi L. (2008). Microscopic analysis of lizard claw morphogenesis and hypothesis on its evolution. Acta Zool..

[75] Alibardi L. (2010). Autoradiographic observations on developing and growing claws of reptiles. Acta Zool..

[76] Byrne C., Hardman M., Nield K. (2003). Covering the limb—Formation of the integument. J. Anat..

[77] Sawyer R.H., Knapp L.W. (2003). Avian skin development and the evolutionary origin of feathers. J. Exp. Zool. B.

[78] Alibardi L. (2003). Adaptation to the land: The skin of reptiles in comparison to that of amphibians and endotherm amniotes. J. Exp. Zool..

[79] Dhouailly D., Maderson P.F.A. (1984). Ultrastructural observations on the embryonic development of the integument of *Lacerta muralis* (Lacertilia, Reptilia). J. Morphol..

[80] Carver W.E., Sawyer R.H. (1987). Development and keratinisation of the epidermis of the common lizard, *Anolis carolinensis*. J. Exp. Zool..

[81] Holthaus K.B., Mlitz V., Strasser B., Tschachler E., Alibardi L., Eckhart L. (2017). Identification and comparative analysis of the epidermal differentiation complex in snakes. Sci. Rep..

[82] Richardson K.C., Park J.Y., Webb J.W., Manolis S.C., Grigg G., Seebacher F., Franklin C.E. (2000). Skin histology of embryonic and hatchling estuarine and australian freshwater crocodiles. Crocodilian Biology and Evolution.

[83] Alibardi L., Thompson M.B. (2001). Fine structure of the developing epidermis in the embryo of the American alligator (*Alligator mississippiensis,* Crocodilia, Reptilia). J. Anat..

[84] Alibardi L., Dipietrangelo L. (2005). Differentiation of the epidermis of scutes in embryos and juveniles of the tortoise *Testudo hermanni* with emphasis on beta-keratinization. Acta Zool..

[85] Wang Z., Pascual-Anaya J., Zadissa A., Li W., Niimura Y., Huang Z., Li C., White S., Xiong Z., Fang D. (2013). The draft genomes of soft-shell turtle and green sea turtle yield insights into the development and evolution of the turtle-specific body plan. Nat. Genet..

[86] Holthaus K.B., Alibardi L., Tschachler E., Eckhart L. (2020). Identification of epidermal differentiation genes of the tuatara provides insights into the early evolution of lepidosaurian skin. Sci. Rep..

[87] Dalla Valle L., Nardi A., Belvedere P., Toni M., Alibardi L. (2007). Betakeratins of differentiating epidermis of snake comprise glycineproline- serine-rich proteins with an avian-like gene organization. Dev. Dynam..

[88] Yenmiş M. (2022). İki Agamid Kertenkelesinin Karşılaştırmalı Ekomorfolojik Analizi: *Stellagama stellio* ve *Trapelus lessonae* Popülasyonlarında Habitat Farklılıklarının Fenotipik Etkisi. Ph.D. Thesis.

[89] Karabinos A., Riemer D., Erber A., Weber K. (1998). Homologues of vertebrate type I, II and III intermediate filaments (IF) proteins in an invertebrate: The IF multigene family of the cephalocordate Branchiostoma. FEBS Lett..

[90] Schaffeld M., Schultess J. (2006). Genes coding for intermediate filament proteins closely related to the hagfish “thread keratins (TK)” alpha and gamma also exist in lamprey, teleosts and amphibians. Exp. Cell Res..

[91] Vanhoutteghem A., Djian P., Green H. (2008). Ancient origin of the gene encoding involucrin, a precursor of the cross-linked envelope of epidermis and related epithelia. Proc. Natl. Acad. Sci. USA.

[92] Martin L.D., Czerkas S.A. (2000). The fossil record of feather evolution in the mesozoic. Am. Zool..

[93] Maddin H.C., Reisz R.R. (2007). The morphology of the terminal phalanges in Permo- Carboniferous synapsids: An evolutionary perspective. Can. J. Earth Sci..

[94] Eckhart L., Dalla Valle L., Jaeger K., Ballaun C., Szabo S., Nardi A., Buchberger M., Hermann M., Alibardi L., Tschachler E. (2008). Identification of reptilian genes encoding hair keratin-like proteins suggests a new scenario for the evolutionary origin of hair. Proc. Natl. Acad. Sci. USA.

[95] Alibardi L. (2012). Immunolocalization of keratin associated beta-proteins (beta-keratins) in the regenerating lizard epidermis indicates a new process for the differentiation of the epidermis in lepidosaurians. J. Morphol..

[96] Alibardi L., Rogers G. (2015). Observations on fur development in echidna (monotremata, Mammalia) indicate that spines precede hairs in ontogeny. Anat. Rec..

[97] Greenwold M.J., Sawyer R.H. (2011). Linking the molecular evolution of avian beta keratins to the evolution of feathers. J. Exp. Zool..

[98] Gregg K., Rogers G., Bereiter-Hahn J. (1986). Feather keratins: Composition, structure and biogenesis. Biology of the Integument, Vertebrates.

